# Effects of Muscarinic Acetylcholine 3 Receptor^208-227^ Peptide Immunization on Autoimmune Response in Nonobese Diabetic Mice

**DOI:** 10.1155/2013/485213

**Published:** 2013-12-09

**Authors:** Lin Yang, Jinzhe Ju, Wei Zhang, Fengfeng Lv, Chunyan Pang, Guoan Yang, Yongfu Wang

**Affiliations:** Key Autoimmunity Lab of Inner Mongolia, Institution of Immunology and Rheumatism, Baotou Medical College, No. 41 Lin yin Road, Baotou 014010, China

## Abstract

The second extracellular loop (LFWQYFVGKRTVPPGECFIQFLSEPTITFGTAI, aa 205–237) of muscarinic acetylcholine 3 receptor (M3R) has been reported to be an epitope for autoantibodies generated during certain autoimmune disorders, including Sjögren's syndrome (SS). Autoantibodies against M3R^228–237^ have been shown to interfere with the function of M3R. However, few studies have been performed on the M3R^205–227^ peptide of the second extracellular loop. In the current study, we sought to investigate the effect of M3R^208–227^ peptide immunization on autoimmune response in NOD/LtJ mice. We synthesized the M3R^208–227^ peptide and immunized NOD/LtJ mice to investigate whether peptide-specific antibodies could be generated and whether immunization would lead to changes in autoimmune response in NOD/LtJ mice. Our results demonstrate that the secretions of Th-1, Th-2, and Th-17 cytokines are downregulated and lymphocytic infiltration is improved in the salivary glands and lacrimal glands following immunization with M3R^208–227^ peptide in NOD/LtJ mice, suggesting that peptide immunotherapy using the M3R^208–227^ peptide may represent a potential therapeutic alternative.

## 1. Introduction

The muscarinic acetylcholine 3 receptor (M3R) plays a key role in mediating exocrine protein secretion in the salivary and lacrimal glands. Autoimmune responses against M3Rs contribute to the development of sicca symptoms and autonomic dysfunction in patients with both primary and secondary Sjögren's syndrome (SS) [1, 2], which suggests that disorders related to M3R signaling in the salivary and lacrimal glands can lead to reduced secretions from these glands.

Iizuka et al. [3] demonstrated that the M3R reactive T helper cells play a crucial role in the pathogenesis of SS. The second extracellular loop (LFWQYFVGKRTVPPGECFIQFLSEPTITFGTAI, aa 205–237) of M3R has been reported to be an epitope for autoantibodies generated during certain autoimmune disorders [4–7], and autoantibodies against M3R^205–237^ have been shown to interfere with the function of M3R [4, 8–10]. Cavill et al. [11] reported that a short peptide sequence of 10 amino acids (EPTITFGTAI, aa 228–237) located at the COOH-terminal region of the second extracellular loop was shown to possess the strongest inhibitory activity, and the inhibitory activity of this short peptide was subsequently confirmed by Koo et al. [12]. However, few studies have been performed on the M3R^205–227^ peptide of the second extracellular loop. The study by Koo et al. showed that the M3R^205–230^ peptide does not bind to autoantibodies from patients with primary SS [12], and a separate study demonstrated that the M3R^213–228^-GST fusion peptide was antigenic only as a dimer [9]. Furthermore, the study by Naito et al. [13] demonstrated that VPPGECFKQFLSEPT (M3R 223I→K) and VPPGECFIAFLSEPT (M3R 224Q→A) for the anchor positions binding to HLA-DR B1*0901 were candidate altered peptide ligands of the second extracellular domain of M3R.

In the current study, we sought to investigate the effect of M3R^208–227^ peptide immunization on autoimmune response in NOD/LtJ mice. We synthesized the M3R^208–227^ peptide and immunized NOD/LtJ mice to investigate whether peptide-specific antibodies could be generated and whether immunization would lead to changes in autoimmune response in NOD/LtJ mice. Our results suggest that the secretions of Th-1, Th-2, and Th-17 cytokines are downregulated and lymphocytic infiltration is improved in the salivary glands and lacrimal glands following immunization with M3R^208–227^ peptide in NOD/LtJ mice, suggesting that peptide immunotherapy using the M3R^208–227^ peptide needs further studies.

## 2. Materials and Methods

### 2.1. Animals

Eight-week-old female NOD/LtJ mice were purchased from the Institute of Laboratory Animal Sciences at CAMS and PUMC (Beijing, China). The animals were maintained in a pathogen-free facility in the Animal Laboratory of Baotou Medical College. All procedures involving animals were performed according to the Research Animal Administration Guidelines of China and the Guidelines for the Care and Use of Laboratory Animals in China.

### 2.2. Peptides and Immunization

Four separate peptides, including the part of second extracellular loop (M3R^208–227^, QYFVGKRTVPPGECFIQFLS), C-terminal of the second extracellular loop (M3R^228–237^, EPTITFGTAI), the third extracellular loop (M3R^514–527^, NTFCDSCIPKTFWN), and the N-terminus of the murine M3R extracellular domain (MTLHSNSTTSPLFPNISSSW), were synthesized chemically via the solid-phase procedure and were purified by high-performance liquid chromatography (GL BioChem, Shanghai, China). The N-terminus of the murine M3R extracellular domain served as a control peptide (CP). NOD/LtJ mice were divided into either the M3R^208–227^, CP, or PBS groups (*n* = 8 per group) and received 100 *μ*g of M3R^208–227^ or CP diluted in PBS and emulsified in complete Freund's adjuvant (CFA; Wako Pure Chemical Industries, Osaka, Japan). Injections of PBS were used as an additional control. Three weeks later, NOD/LtJ mice received another injection of M3R^208–227^ or CP diluted in PBS and emulsified in incomplete Freund's adjuvant (IFA; Wako Pure Chemical Industries, Osaka, Japan).

### 2.3. Antibody Titer Determination

The sera were collected every seven days after the second immunization. Ninety six well ELISA plates (JCHS, Shenzhen, China) were coated with M3R^208–227^, M3R^228–237^, and M3R^514–527^ peptide solutions (10 *μ*g/mL in 0.1 M NaHCO_3_ (pH 8.6)) overnight at 4°C and were then blocked with 0.05% Tween 20 in PBS containing 5% dry milk for 2 h at 37°C. Sera diluted 1 : 50 in blocking buffer were incubated for 2 h at 37°C. The plates were then washed six times with 0.05% Tween 20 in PBS containing 5% dry milk, and an HRP-conjugated anti-mouse IgG antibody (Abcam, Cambridge, UK) diluted 1 : 1000 in blocking buffer was added for 1 h at 37°C. After washing, the reaction was developed with 2,2,-azino-bis-(3-ethylbenzthiazoline-6-sulfonic acid) (Sigma, St. Louis, MO) as a substrate. The optical density at 405 nm was measured using an ELISA reader (BioRad model 680).

### 2.4. Analysis of Cytokines in the Sera and Cell Supernatants

The levels of various cytokines in the sera were evaluated after the second immunization according to the instructions provided by the ELISA kits (IL-17, TGF-*β*_1_, and IFN-*γ*: eBioscience, San Diego, CA; IL-4, IL-10, and IL-6: RayBiotech, Norcross, GA). The serum samples were diluted 1 : 50.

The spleen cells were obtained from the mice sacrificed in each group four weeks after the second immunization. Homogenized spleens from NOD/LtJ mice immunized with the M3R^208–227^ peptide were prepared as described previously with some modifications [3]. Following serum collection, the mice were sacrificed, and their spleens were isolated and pooled. Red blood cells were removed by treatment with a 0.16 M NH_4_Cl solution. Then, the homogenates were adjusted to 1 × 10^6^ cells/mL and incubated with RPMI-1640 supplemented with 10% FBS (Gibco, Grand Island, NE), 1000 U/mL penicillin, 100 mg/mL streptomycin, and M3R^208–227^, CP, or PBS in an atmosphere of 5% CO_2_ at 37°C. The cell supernatants were obtained after 48 hours, at which time the levels of the various cytokines were measured using ELISA kits (IL-17, TGF-*β*_1_, and IFN-*γ*: eBioscience, San Diego, CA; IL-4, IL-10, and IL-6: RayBiotech, Norcross, GA).

### 2.5. Histology

The salivary glands and lacrimal glands were obtained from the mice sacrificed in each group four weeks after the second immunization. Following sacrifice, the entire lacrimal glands of NOD/LtJ mice were surgically removed and placed in 10% phosphate-buffered formalin for 20 min. Paraffin-embedded sections were deparaffinized by immersion in xylene, followed by dehydration in ethanol. The tissue sections were prepared and stained with hematoxylin and eosin (H&E). Sections were observed at a 200x magnification under an Olympus IX71 microscope (Olympus, Shanghai, China). Images were obtained with DP2-BSW software (Olympus, Shanghai, China). Leukocytic infiltrations were analyzed with Image-Pro Plus 6.0 software (Media Cybernetics) in the histological sections of lacrimal glands. The lymphocytic infiltration (histological score) was graded using the method proposed by Greenspan et al. [14].

Immunofluorescence staining was performed as described previously with some modifications [3]. The sections were incubated with rat anti-mouse IFN-*γ*, rat anti-mouse IL-4, and rat anti-mouse IL-17 antibodies (Santa Cruz Biotechnology, CA) for 30 min. To detect primary antibodies, Alexa Fluor 546 goat anti-rat IgG antibody (Invitrogen, Carlsbad, CA) was added for 30 min. Sections were observed at a 100x magnification under an Olympus IX71 microscope (Olympus, Shanghai, China). Images were obtained with DP2-BSW software (Olympus, Shanghai, China).

### 2.6. Statistical Analysis

The data are expressed as the mean ± SD. The images were analyzed with Image-Pro Plus 6.0 software. Differences between groups were evaluated for statistical significance using the Student's *t*-test, and *P* values <0.05 were considered statistically significant.

## 3. Results

### 3.1. Generation of Peptide-Specific Antibodies

**Figure 1:**
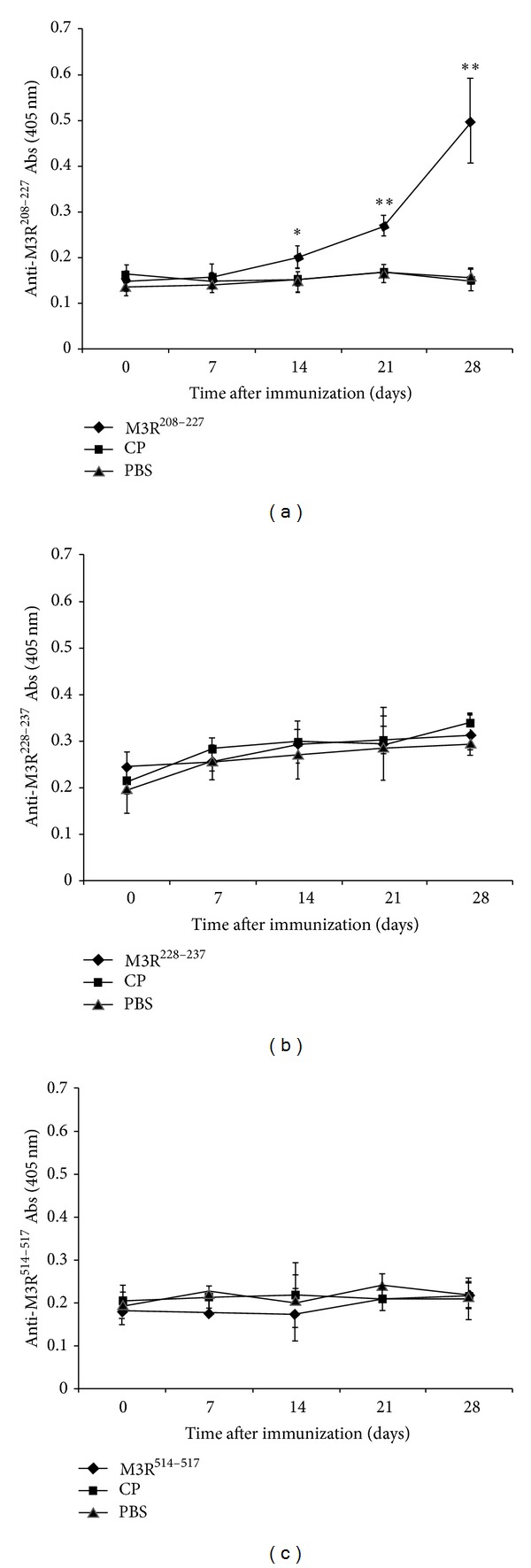
Immunization with the M3R^208–227^ peptide induced peptide-specific antibodies. NOD/LtJ mice received 100 *μ*g M3R^208–227^ (rhombus) or CP (square) diluted in PBS and emulsified in incomplete Freund's adjuvant. PBS immunizations (triangle) were used as an additional control. Sera were obtained from the mice in each group every seven days after the second immunization, and these samples were diluted 1 : 50. Antibody titer determination was performed by ELISA. The results showed that peptide-specific antibodies were successfully induced in NOD/LtJ mice immunized with the M3R^208–227^ peptide (a), but no changes were observed in antibody titers against M3R^228–237^ (C-terminal) (b) and M3R^514–527^ (the third extracellular loop) (c) in M3R^208–227^ peptide immunized NOD/LtJ mice (mean ± SD; *P < 0.05; **P < 0.005).

We performed ELISAs to determine whether peptide-specific antibodies could be detected in the sera after the second immunization. As shown in Figure 1, the serum titers of anti-M3R^208–227^ antibodies were higher in M3R^208–227^ peptide immunized NOD/LtJ mice than in the CP and PBS control groups, but no changes were observed in antibody titers against M3R^228–237^ and M3R^514–527^ in M3R^208–227^ peptide immunized NOD/LtJ mice (Figures 1(a), 1(b), and 1(c)). These results showed that peptide-specific antibodies were successfully and specifically generated only in animals immunized with the M3R^208–227^ peptide.

### 3.2. Reductions in the Levels of Cytokines in the Sera

**Figure 2:**
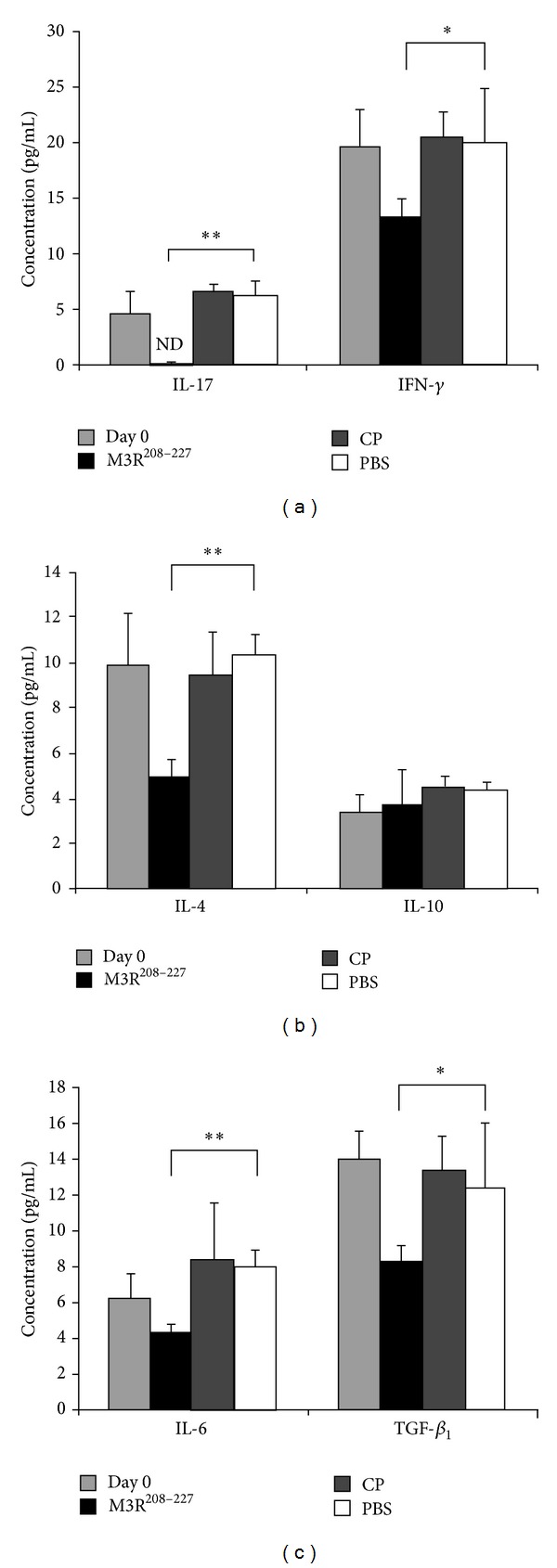
Downregulated expression of IFN-*γ*, IL-4, IL-17, IL-6, and TGF-*β*_1_ in the serum following immunization with the M3R^208–227^ peptide. Sera were collected from mice in each group before immunization (light gray bar), and four weeks following the second immunization with M3R^208–227^ (black bar), CP (charcoal gray bar), or PBS (white bar), and the samples were diluted 1 : 50. The cytokine levels were measured using ELISA kits. (a) The results showed that the concentration of IFN-*γ* was significantly decreased in the sera from mice immunized with M3R^208–227^ in comparison to those in the sera of the control animals. On the other hand, the concentration of IL-17 was below the detection level (*P < 0.05; **P < 0.005). (b) These results showed that the concentration of IL-4 was significantly decreased in the sera of M3R^208–227^ peptide immunized mice as compared to the control groups (**P < 0.005). No significant decrease in IL-10 secretion was observed (*P* > 0.05). (c) The results demonstrated that the concentrations of TGF-*β*_1_ and IL-6 were significantly decreased in the sera from mice immunized with M3R^208–227^ as compared to the controls (*P < 0.05; **P < 0.005).

Following the determination of antibody titers, the specificity of the Th-produced cytokines present in the sera was evaluated by comparing the cytokine profiles of animals immunized with the M3R^208–227^ peptide to those of animals immunized with CP or PBS. The cytokine concentrations were measured by ELISA. First, we measured the Th-17-associated cytokine IL-17 and the Th-1-associated cytokine IFN-*γ*, which are known to play crucial roles in the development of autoimmune disease. The results showed that the concentration of IFN-*γ* in the serum decreased following immunization with M3R^208–227^ as compared to the control groups. Meanwhile, the concentration of IL-17 was below the detection level (IL-17, *P* < 0.005; IFN-*γ*, *P* < 0.05) (Figure 2(a)).

Consequently, we analyzed the levels of Th-2-associated cytokines in the sera of NOD/LtJ mice. IL-4 is thought to inhibit the production of IFN-*γ* by Th-1 cells, and IL-10 is also an inhibitory cytokine, but recent research has shown that both of these cytokines are positively correlated with the pathogenic process of SS. Our data showed that immunization with the M3R^208–227^ peptide resulted in a significant reduction in IL-4, as compared to the control groups (*P* < 0.005). However, no trend toward decreased concentrations of IL-10 was observed (*P* > 0.05) (Figure 2(b)).

Moreover, recent data have shown that the levels of TGF-*β*_1_ mRNA transcripts and protein were significantly higher in animals with an experimental model of dry eye [15]. As TGF-*β*_1_ can synergize with IL-6 to induce the upregulation of the transcription factor ROR*γ*t, which promotes Th-17 differentiation and the secretion of IL-17 [16–19], we also evaluated the levels of IL-6 and TGF-*β*_1_. These results demonstrated that both IL-6 and TGF-*β*_1_ were downregulated in the sera of NOD/LtJ mice immunized with M3R^208–227^, which further confirmed the important role for IL-6 and TGF-*β* in Th-17 differentiation (IL-6, *P* < 0.005; TGF-*β*_1_, *P* < 0.05) (Figure 2(c)).

### 3.3. Reductions in the Levels of Cytokines in Cell Supernatants

**Figure 3:**
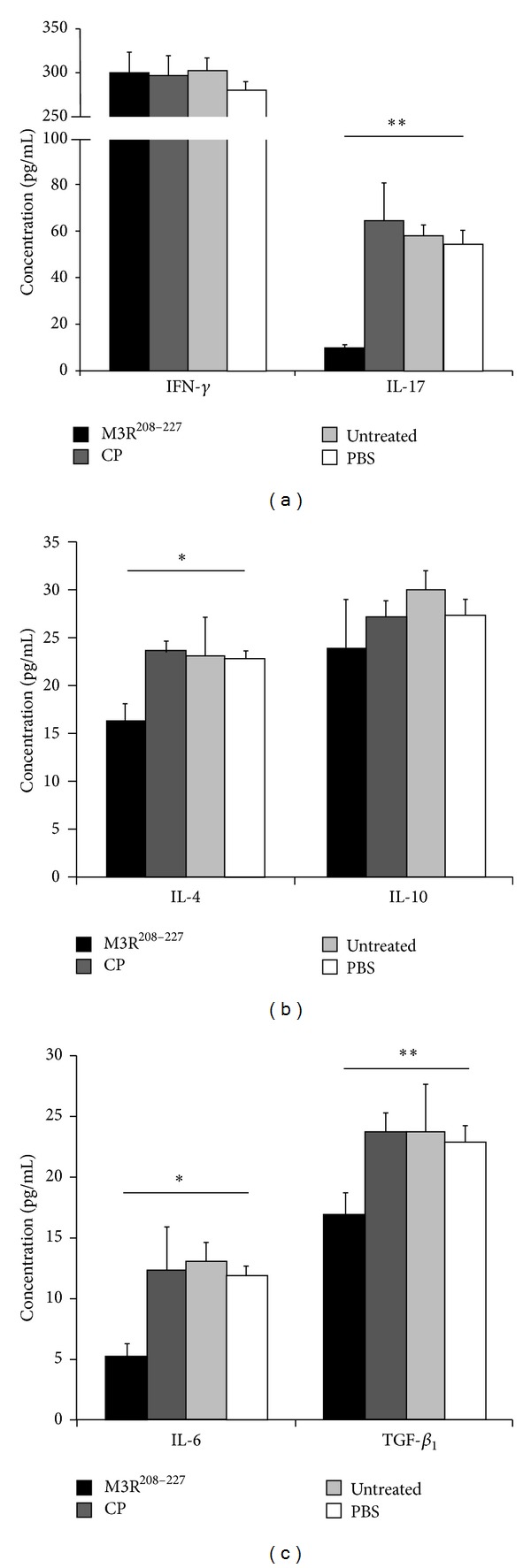
The secretions of IL-17, IL-4, IL-6, and TGF-*β*_1_ were decreased in the cell supernatants following incubation with M3R^208–227^ peptide. Spleens were isolated from the NOD/LtJ mice immunized with the M3R^208–227^ peptide and the spleen cells were pooled. The homogenates were adjusted to 1 × 10^6^ cells/mL and incubated with M3R^208–227^ (black bar), CP (charcoal gray bar), or PBS (white bar). Untreated spleen cells served as an additional control (light gray bar). Cell supernatants were collected after 48 hours of culture, and the cytokine concentrations were measured using ELISA kits. (a) The results indicated that the secretion of IL-17 was significantly reduced in the supernatants of cells that had been cocultured with the M3R^208–227^ peptide in comparison to those cocultured with CP and PBS or left untreated (**P < 0.005), although no significant decrease in IFN-*γ* was observed (*P* > 0.05). (b) These results showed that the secretion of IL-4 was also significantly decreased in the supernatants of cells cocultured with M3R^208–227^ as compared to those cocultured with CP and PBS or left untreated (*P < 0.05). No significant decrease in IL-10 secretion was observed (*P* > 0.05). (c) The results showed that the secretion of IL-6 and TGF-*β*_1_ was significantly decreased in the supernatants of cells cocultured with M3R^208–227^ as compared to those cocultured with CP and PBS or left untreated (*P < 0.05; **P < 0.005).

Here, we assessed the cytokine profiles of cell culture supernatants after spleen cells were cocultured with either the M3R^208–227^ peptide, CP, or PBS. Untreated spleen cells were used as an additional negative control. The results showed that the secretion of IL-17 in cell culture supernatant decreased following immunization with M3R^208–227^ as compared to the control groups (*P* < 0.005). However, in cell culture supernatant, no significant decrease in IFN-*γ* secretion was observed (*P* > 0.05) (Figure 3(a)). Our data also showed that immunization with the M3R^208–227^ peptide resulted in a significant reduction in IL-4 in cell culture supernatant, as compared to the control groups (*P* < 0.05). No trend toward decreased concentrations of IL-10 was observed (*P* > 0.05) (Figure 3(b)). The changes in the secretions of IL-6 and TGF-*β*_1_ were also downregulated in cell culture supernatants of NOD/LtJ mice immunized with M3R^208–227^ compared to the control groups (IL-6, *P* < 0.005; TGF-*β*_1_, *P* < 0.05) (Figure 3(c)).

### 3.4. Reductions in the Levels of IL-17 and IFN-*γ* in the Lacrimal Glands

**Figure 4:**
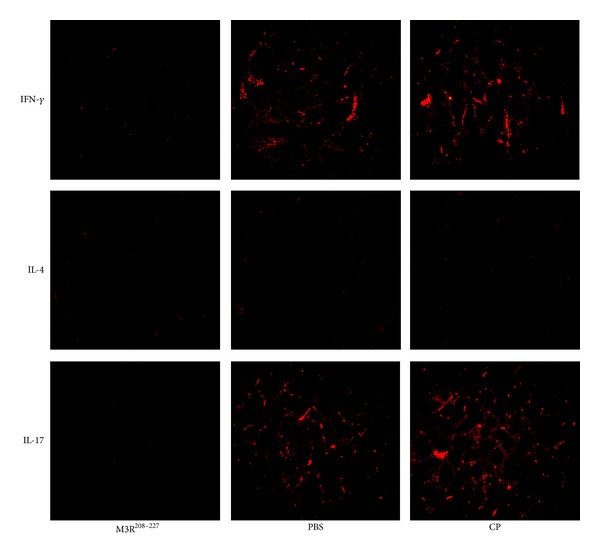
Reductions in the levels of IL-17 and IFN-*γ* in the lacrimal glands following immunization with the M3R^208–227^ peptide. To further observe whether the concentrations of cytokines decreased in the lacrimal glands, we also examined the production of IFN-*γ*, IL-4, and IL-17 in the lacrimal glands of NOD/LtJ mice by immunofluorescence staining. Whole lacrimal glands from NOD/LtJ mice were removed, fixed, embedded, and incubated with primary antibodies, followed by a secondary goat anti-rat IgG antibody. The results of immunofluorescence staining demonstrated that the production of IL-17 and IFN-*γ* decreased in the lacrimal glands of NOD/LtJ mice immunized with the M3R^208–227^ peptide. In contrast, the production of IL-4 was not observed in the lacrimal glands of NOD/LtJ mice in all groups.

To further observe whether the concentrations of cytokines decreased in the lacrimal glands, we also examined the production of IFN-*γ*, IL-4, and IL-17 in the lacrimal glands of NOD/LtJ mice by immunofluorescence staining. Whole lacrimal glands from NOD/LtJ mice were removed, fixed, embedded, and incubated with primary antibodies, followed by a secondary goat anti-rat IgG antibody. The result of immunofluorescence staining showed that the production of IL-17 and IFN-*γ* decreased in the lacrimal glands of NOD/LtJ mice immunized with the M3R^208–227^ peptide. However, the production of IL-4 was not observed in the lacrimal glands of NOD/LtJ mice in all groups (Figure 4).

### 3.5. Improved Lymphocytic Infiltration in the Salivary Glands and Lacrimal Glands

**Figure 5:**
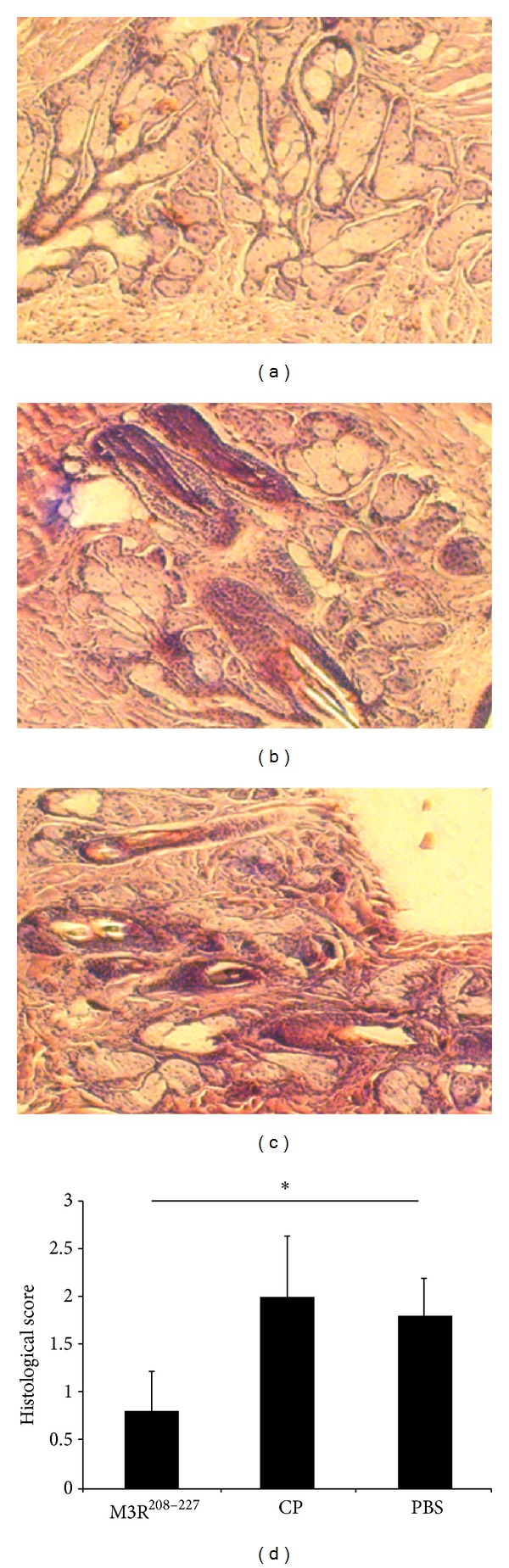
Lymphocytic infiltration was improved in the salivary glands following immunization with the M3R^208–227^ peptide. Whole salivary glands of NOD/LtJ mice were surgically removed from each mouse and placed in 10% phosphate-buffered formalin for 20 min. After the samples were fixed, embedded, and stained with hematoxylin and eosin (H&E), sections were observed under a microscope. The histological analysis suggested that immunization with M3R^208–227^ could reduce the lymphocytic infiltration in the salivary glands of NOD/LtJ mice. (a) Tissue from a NOD/LtJ mouse immunized with the M3R^208–227^ peptide. (b) Tissue from a NOD/LtJ mouse immunized with CP. (c) Tissue from a NOD/LtJ mouse immunized with PBS. (d) Mean grade (histological score) of inflammatory lesions in salivary glands of NOD/LtJ mouse immunized with the M3R^208–227^ peptide as compared to the controls (*P < 0.05).

**Figure 6:**
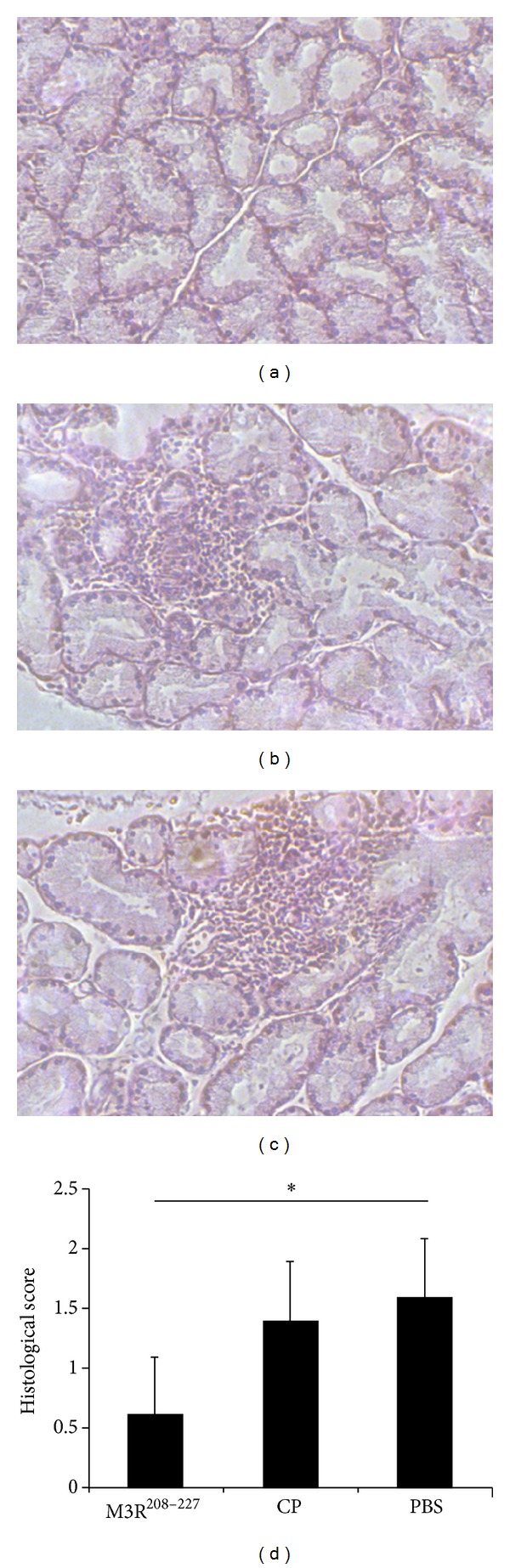
Lymphocytic infiltration was improved in the lacrimal glands following immunization with the M3R^208–227^ peptide. Whole lacrimal glands of NOD/LtJ mice were surgically removed from each mouse and placed in 10% phosphate-buffered formalin for 20 min. After the samples were fixed, embedded, and stained with hematoxylin and eosin (H&E), sections were observed under a microscope. The histological analysis suggested that immunization with M3R^208–227^ could reduce the lymphocytic infiltration in the lacrimal glands of NOD/LtJ mice. (a) Tissue from a NOD/LtJ mouse immunized with the M3R^208–227^ peptide. (b) Tissue from a NOD/LtJ mouse immunized with CP. (c) Tissue from a NOD/LtJ mouse immunized with PBS. (d) Mean grade (histological score) of inflammatory lesions in lacrimal glands of NOD/LtJ mouse immunized with the M3R^208–227^ peptide as compared to the controls (*P < 0.05).

To evaluate lymphocytic infiltration in the salivary glands and lacrimal glands, whole salivary glands and lacrimal glands from NOD/LtJ mice were removed, fixed, embedded, and stained with hematoxylin and eosin (H&E). Lymphocytic infiltration was decreased in the salivary glands and lacrimal glands of NOD/LtJ mice immunized with M3R^208–227^. In contrast, marked lymphocytic infiltration was observed in the salivary glands (Figure 5) and lacrimal glands (Figure 6) of animals in the control groups (*P* < 0.05).

## 4. Discussion

Elias et al. [20] demonstrated that pep277, a peptide of the human 60 kDa heat-shock protein, was found to be a therapeutic agent to arrest the autoimmune process in NOD mice. In addition, He et al. [21] demonstrated that mucosal administration of *α*-fodrin, a 120 kd autoantigen in Sjögren's syndrome, effectively inhibited the progression of experimental Sjögren's syndrome autoimmunity. In this study, we aimed to evaluate the effect of M3R^208–227^ peptide immunization on autoimmune response of NOD/LtJ mice. As immunization with M3R^208–227^ peptide conjugated to an immunogenic carrier protein such as KLH may induce some uncertain immunological responses and immunization with the entire second extracellular loop tends to be pathogenic and, in addition, Naito et al. [13] demonstrated that VPPGECFKQFLSEPT (M3R 223I→K) and VPPGECFIAFLSEPT (M3R 224Q→A) were candidate altered peptide ligands of the second extracellular domain of M3R, we immunized NOD/LtJ mice intradermally with the M3R^208–227^peptide. The ELISA results showed that anti-M3R^208–227^ antibodies were induced successfully and specifically in M3R^208–227^ immunized animals as compared to the CP and PBS groups. Autoantibodies against M3R^228–237^ and M3R^514–527^ were reported in patients with Sjögren's syndrome [10, 11], but no changes were observed in antibody titers against M3R^228-237^ and M3R^514–527^ in M3R^208–227^ peptide immunized NOD/LtJ mice, suggesting that generations of autoantibodies against M3R^228–237^ and M3R^514–527^ may not be regulated by immunization with M3R^208–227^ peptide in NOD/LtJ mice.

The IFN-*γ* and IL-17 have been shown to play crucial roles in the pathogenesis of SS [22–27]. Therefore, we measured the concentrations of IL-17 and IFN-*γ* in the sera, cell supernatants, and lacrimal glands from M3R^208–227^ immunized mice and found that these concentrations were decreased in comparison to the control-immunized mice. This decrease in the levels of IL-17 and IFN-*γ* suggested that the activities of the secretions of IL-17 and IFN-*γ* were inhibited following M3R^208–227^ immunization in NOD/LtJ mice.

Then, we evaluated the Th-2 cytokines IL-4 and IL-10 and found that the activity of IL-4 was significantly decreased in the serum and cell culture supernatants of M3R^208–227^ immunized mice, although decreases in IL-10 were not observed. One important function of IL-4 is to promote the differentiation of CD4^+^ T cells into Th-2 cells, which antagonizes the production of IFN-*γ* by Th-1 cells. However, recent data have shown that IL-4 was required to regulate the synthesis of pathogenic autoantibodies against M3R by B lymphocytes in the NOD.IL-4-gene knockout mouse model of Sjögren's syndrome [28]. In the current study, the production of IL-4 was not observed in the lacrimal glands of NOD/LtJ mice in all groups, suggesting that IL-4 may be more required to regulate the B-cell autoimmune response in this animal model. IL-10 is a cytokine capable of inhibiting the synthesis of other proinflammatory cytokines, but the secretion of IL-10 has also been shown to be elevated in patients with SS [29, 30]. However, no changes in the concentration of IL-10 were observed in the current study, suggesting that IL-10 may not be regulated following immunization with M3R^208–227^.

The lymphocytic infiltration in the salivary glands and lacrimal glands in M3R^208–227^ treated mice compared to control peptide and PBS was measured four weeks after the second immunization. Jonsson et al. [31] demonstrated that salivary gland inflammation does not necessarily correlate with salivary flow, so we graded the lymphocytic infiltration by using histological score instead of measurement of salivary flow. Lower histological scores showed that tissue from the M3R^208–227^ immunized mice revealed at that time point less infiltrates in the salivary glands and lacrimal glands after immunization, suggesting that the effect of M3R^208–227^ immunization may induce a delay in disease symptoms in NOD/LtJ mice.

The secretions of Th-1, Th-2, and Th-17 cytokines and leukocyte infiltration may have been reduced in the NOD/LtJ mice after immunization with M3R^208–227^. These changes in cytokine secretion and leukocyte infiltration observed could be interpreted in many different ways. The most likely explanation could be that the administration of M3R^208–227^ induced some other antigen-specific T or B regulatory cells that secreted other cytokines which could have immunomodulatory activity. Antigen-specific regulatory cells may suppress autoimmune response by inhibiting the generation of Th-1, Th-2, and Th-17 cells. In addition, the study by Tsuboi et al. [32] indicated that the presence of several different B-cell epitopes within M3R could influence salivary secretion, so it is possible that peptide-specific antibodies may also relate to this process.

In conclusion, immunization with the M3R^208–227^ peptide reduced the secretions of Th-1, Th-2, and Th-17 cytokines and leukocyte infiltration in NOD/LtJ mice, suggesting that immunotherapy with the M3R^208-227^ peptide needs further studies.
